# Phenotypic screening of hypervirulent *Klebsiella pneumoniae* using tellurite resistance and carbohydrate fermentation testing

**DOI:** 10.1128/spectrum.01838-26

**Published:** 2026-08-17

**Authors:** Koji Ohyama, Akiko Sei, Takuya Hosoda, Sohei Harada, Christian Francisco, Angelo dela Tonga, Janet S. Lee, Yohei Doi, Masahiro Suzuki

**Affiliations:** 1 Department of Microbiology, Fujita Health University School of Medicine, Aichi, Japan; 2 Department of Microbiology and Infectious Diseases, Toho University School of Medicine, Tokyo, Japan; 3 Institute of Molecular Biology and Biotechnology, National Institutes of Health, University of the Philippines Manila 54725, Manila, Philippines; 4 Division of Pulmonary and Critical Care Medicine, John T. Milliken Department of Medicine, Washington University in St. Louis School of Medicine 12275, St. Louis, Missouri, USA; 5 Department of Infectious Diseases, Fujita Health University School of Medicine, Aichi, Japan; 6 Center for Innovative Antimicrobial Therapy, Division of Infectious Diseases, University of Pittsburgh School of Medicine 12317, Pittsburgh, Pennsylvania, USA; Cincinnati Children's Hospital Medical Center, Cincinnati, Ohio, USA

**Keywords:** hypervirulent, *Klebsiella pneumoniae*, ST23, tellurite, adonitol, dulcitol, L-sorbose, fermentation, phenotypic screening

## Abstract

Phenotypic identification of hypervirulent *Klebsiella pneumoniae* in routine laboratories remains challenging. We evaluated tellurite resistance and selected carbohydrate fermentation tests as phenotypic tools for screening hypervirulent strains and improving lineage identification. A total of 193 *K. pneumoniae* species complex strains with available whole-genome sequencing data were analyzed, including *K. pneumoniae* (*n* = 152), *K. variicola* (*n* = 29), and *K. quasipneumoniae* (*n* = 12). Hypervirulent strains were defined as those carrying ≥4 of 5 virulence-associated genes (*iucA*, *iroB*, *peg-344*, *rmpA*, and *rmpA2*). A total of 68 strains (35.2%) were classified as hypervirulent, and 74 (38.3%) carried *ter* genes. Hypervirulent strains were strongly associated with *ter* gene carriage, and the tellurite-resistant phenotype on MacConkey agar containing 4 μg/mL potassium tellurite closely matched *ter* gene carriage. Among tellurite-resistant strains, adonitol non-fermentation was useful for excluding non-hypervirulent *K. variicola*. Using combined tellurite resistance and adonitol fermentation, the assay identified hypervirulent strains with a sensitivity of 86.8%, specificity of 89.6%, and accuracy of 88.6%, all higher than the string test. All 27 ST23 strains showed dulcitol fermentation and l-sorbose non-fermentation. Adonitol fermentation improved screening performance, whereas dulcitol and l-sorbose fermentation patterns supported presumptive identification of prototypical hypervirulent lineages. These findings support the potential utility of this culture-based approach for phenotypic screening of hypervirulent *K. pneumoniae* in clinical microbiology laboratories.

**IMPORTANCE:**

Hypervirulent *Klebsiella pneumoniae* is associated with severe invasive infections, but practical methods for early recognition in routine clinical laboratories remain limited. Leveraging the strong association between hypervirulence genotype and *ter* gene carriage, we developed a simple culture-based screening approach for the early presumptive identification of hypervirulent *K. pneumoniae*. This approach, based on combined tellurite resistance and adonitol fermentation, shows higher sensitivity and specificity than the string test for identifying hypervirulent strains. Additionally, dulcitol and l-sorbose fermentation patterns support presumptive identification of prototypical hypervirulent lineages, including ST23. These phenotypic assays may expedite the identification of hypervirulent *K. pneumoniae* and selected hypervirulent lineages in clinical microbiology laboratories.

## INTRODUCTION

Hypervirulent *Klebsiella pneumoniae* has emerged over the past several decades as a major cause of community- and healthcare-associated invasive infections, including pyogenic liver abscess, bacteremia, meningitis, and endophthalmitis (1). Unlike classical *K. pneumoniae*, which primarily affects immunocompromised or hospitalized patients, hypervirulent *K. pneumoniae* can cause severe, disseminated disease in apparently healthy individuals (2). The global spread of hypervirulent *K. pneumoniae*, together with the increasing convergence of hypervirulence and antimicrobial resistance, underscores the urgent need for rapid and reliable methods to identify these strains in routine clinical laboratories (3). Although the *K. pneumoniae* species complex includes closely related species such as *K. variicola* and *K. quasipneumoniae*, the clinical significance of hypervirulence has been most clearly established in *K. pneumoniae* (1, 4, 5).

Molecular studies have identified several plasmid- and chromosome-associated virulence determinants strongly associated with the hypervirulent phenotype. These include the aerobactin siderophore gene cluster (*iuc*), the salmochelin cluster (*iro*), *peg-344*, and the mucoid regulators *rmpA* and *rmpA2* (4, 6). Combinations of these biomarkers have been shown to provide high sensitivity for identifying hypervirulent *K. pneumoniae* (7). However, detection of these genes requires whole-genome sequencing (WGS) or targeted molecular assays, which are not universally available or affordable in routine diagnostic settings, particularly in resource-limited regions where these infections may be common. Accordingly, there is a need for simple phenotypic approaches that can serve as practical surrogates for hypervirulence-associated genotypes.

The string test, based on hypermucoviscosity, has historically been used as a screening tool for hypervirulent *K. pneumoniae* in clinical laboratories (8, 9). However, its diagnostic performance is suboptimal across studies, as hypermucoviscosity does not always correlate with the presence of key virulence genes, and some classical, non-hypervirulent strains may produce positive results (10, 11). More reliable phenotypic markers linked to the underlying genetic determinants are therefore desirable.

Tellurite resistance, mediated by the *ter* operon, has been reported to be strongly associated with hypervirulent lineages, particularly clonal group 23 (CG23), which includes sequence type 23 (ST23), the prototypical hypervirulent lineage (12). The *ter* locus is frequently co-located with virulence-associated loci on plasmids and may serve as a surrogate marker for hypervirulence-associated genetic backgrounds (13, 14). Incorporation of potassium tellurite into selective media may therefore enable phenotypic detection of strains carrying the *ter* operon (12, 15). In addition, carbohydrate utilization patterns have historically been used to differentiate bacterial species and lineages, including sorbitol non-fermentation as a classic phenotypic trait used to screen for *Escherichia coli* O157:H7 (16). In *Klebsiella* species, carbohydrate utilization patterns have also been used to differentiate species and lineages (17). For example, adonitol fermentation testing has been reported as a phenotypic approach for distinguishing *K. variicola* from *K. pneumoniae* (18, 19). However, metabolic features associated with hypervirulent lineages have not been systematically characterized, and their utility for phenotypic identification remains uncertain.

In this study, we evaluated tellurite resistance and selected carbohydrate fermentation tests as phenotypic tools for screening hypervirulent *K. pneumoniae* and improving lineage identification. We first determined the optimal screening conditions for discriminating between *ter*-positive and *ter*-negative strains and assessed a tellurite-based phenotypic strategy for identifying hypervirulent strains. We then evaluated carbohydrate fermentation patterns as supplementary phenotypic markers to improve screening performance and support lineage identification. By combining tellurite resistance with selected carbohydrate fermentation tests, we aimed to develop a practical culture-based approach suitable for phenotypic screening of hypervirulent *K. pneumoniae* in clinical microbiology laboratories.

## MATERIALS AND METHODS

### Selection of strains

We included *K. pneumoniae* species complex isolates collected in Japan between November 2013 and April 2025. A total of 742 strains for which WGS data were available as of 28 May 2025 were included, comprising 334 clinical strains and 408 stool strains recovered from apparently healthy individuals, as previously described (20). Among these strains, all strains carrying ≥3 of the following genes identified by WGS were selected for inclusion: *iucA*, *iroB*, *peg-344*, *rmpA,* and *rmpA2*. The remaining strains were randomly selected from those carrying fewer than three of these genes after limiting inclusion to one strain per patient, yielding an initial study set of 200 strains. Strains differing by ≤10 core-genome single-nucleotide polymorphisms (SNPs) were excluded, and the strain assigned a smaller study identification number was retained when such pairs were identified. This resulted in the final study set of 193 strains.

### MacConkey agar supplemented with potassium tellurite and adonitol

**Fig 1:**
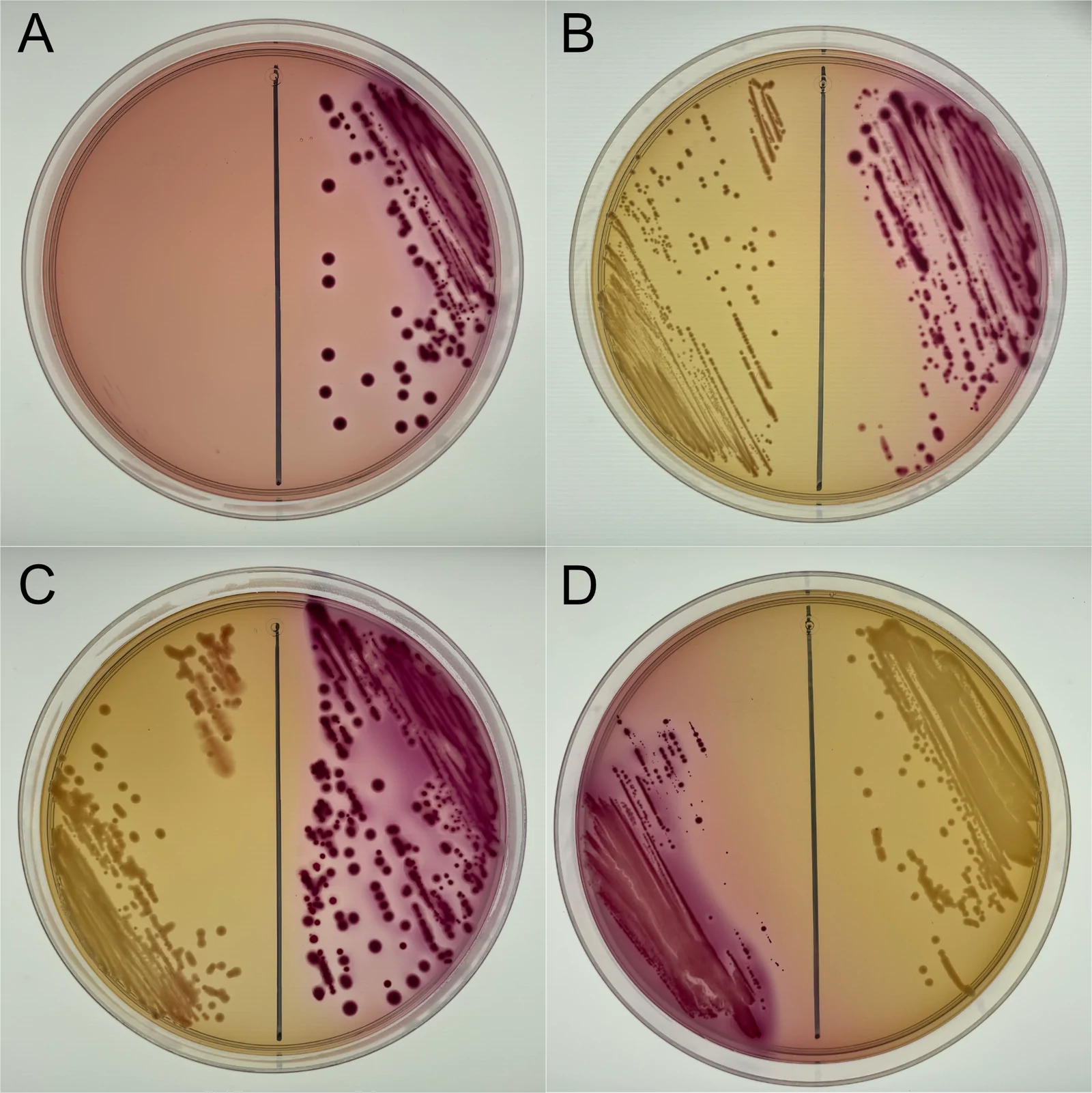
Representative colony phenotypes on MacConkey agar supplemented with tellurite, adonitol, dulcitol, or l-sorbose. Representative colony phenotypes of the *Klebsiella pneumoniae* species complex on selective and differential media. Panels A and B show colony phenotypes on tellurite–adonitol MacConkey agar. (**A**) Left, tellurite-susceptible *K. pneumoniae*; right, tellurite-resistant *K. pneumoniae* with adonitol fermentation, showing pink colonies. (**B**) Left, tellurite-resistant *K. variicola* with adonitol non-fermentation, showing white colonies; right, tellurite-resistant *K. pneumoniae* with adonitol fermentation, showing pink colonies. (**C**) Dulcitol fermentation phenotypes, showing dulcitol non-fermentation with white colonies on the left and dulcitol fermentation with pink colonies on the right. (**D**) l-sorbose fermentation phenotypes, showing l-sorbose fermentation with pink colonies on the left and l-sorbose non-fermentation with white colonies on the right.

MacConkey agar base powder (Becton, Dickinson and Company; catalog no. 281810) was prepared according to the manufacturer’s instructions. Adonitol was added as the carbohydrate source at a final concentration of 1% to assess adonitol fermentation among strains of the *K. pneumoniae* species complex as a supplementary phenotypic marker to improve screening performance by distinguishing *K. pneumoniae* from *K. variicola*. To determine the concentration of potassium tellurite to be used in the main experiment, preliminary testing was performed using MacConkey agar supplemented with potassium tellurite at final concentrations of 1, 2, 4, 8, 16, 50, 100, and 300 μg/mL. This pilot experiment was performed using 30 *K. pneumoniae* species complex strains selected from the final study set of 193 strains, including both *ter*-positive and *ter*-negative strains. Strains of the *K. pneumoniae* species complex stored at −80°C were recovered on 5% sheep blood agar plates. After overnight incubation, colonies were picked directly from the blood agar plates with an inoculating loop and streaked onto the prepared MacConkey agar plates, followed by incubation at 37°C for 18–24 h. The inhibitory concentration of potassium tellurite on agar was defined as the lowest concentration that completely inhibited visible growth under these experimental conditions. Based on the results of this preliminary testing, the concentration of potassium tellurite used for the main experiment was set at 4 μg/mL. Visible growth on MacConkey agar supplemented with potassium tellurite at 4 μg/mL was interpreted as a tellurite-resistant phenotype. Pink colonies were interpreted as indicating adonitol fermentation, whereas white colonies were interpreted as indicating adonitol non-fermentation (Fig. 1).

### Carbohydrate fermentation testing and MacConkey agar supplemented with dulcitol or l-sorbose

To explore carbohydrate fermentation patterns potentially associated with hypervirulent *K. pneumoniae*, the API 50 CH (bioMérieux) was performed according to the manufacturer’s instructions using 10 *K. pneumoniae* strains selected to include hypervirulent strains and non-hypervirulent strains representing diverse sequence types. A positive reaction was defined as acidification of the medium, indicated by a yellow color change of the phenol red indicator. Based on the results of this preliminary screening, dulcitol and l-sorbose were selected for further evaluation on solid medium. MacConkey agar base medium was prepared according to the manufacturer’s instructions, and dulcitol or l-sorbose was added at a final concentration of 1% as the carbohydrate source. For each strain, colonies were picked directly with an inoculating loop, streaked onto the prepared MacConkey agar plates, and incubated at 37°C for 18–24 h. On this medium, pink colonies were interpreted as indicating fermentation of the test carbohydrate, whereas white colonies were interpreted as indicating non-fermentation (Fig. 1).

### String test

The string test was performed using colonies grown overnight on sheep blood agar plates at 37°C. The test was considered positive when a viscous string of ≥5 mm was formed by stretching a colony with an inoculating loop (8).

### Whole-genome sequencing

Genomic DNA was extracted from strains using the Puregene Cell Kit (Qiagen, Hilden, Germany). DNA libraries were prepared with the QIAseq FX DNA Library Kit (Qiagen). WGS was performed using the NextSeq 2000 platform (Illumina, San Diego, CA, USA) with 2 × 150 bp paired-end reads. *De novo* genome assembly was performed using SPAdes v3.13.1. Virulence genes and tellurite resistance genes were detected using AMRFinderPlus v4.0.19 (21). Species identification was performed using FastANI with a 95% average nucleotide identity cutoff based on whole-genome comparisons with reference genomes (Table S1) (22). Multilocus sequence typing (MLST) was conducted *in silico* with the Institut Pasteur scheme using mlst v2.18.0, available from the software repository (https://github.com/tseemann/mlst). Kleborate v3.2.4 was also used to characterize additional genomic features (23). Core-genome SNP analysis was performed using Snippy v4.6.0. A maximum-likelihood phylogenetic tree was reconstructed from the core SNP alignment using RAxML-NG v1.2.2 (24), and the resulting tree was visualized with the Interactive Tree Of Life (iTOL) (25).

### Definition of hypervirulent strains within the *K. pneumoniae* species complex

Strains belonging to the *K. pneumoniae* species complex and harboring ≥4 of the following biomarkers were defined as hypervirulent: *iucA*, *iroB*, *peg-344*, *rmpA*, and *rmpA2*. This cutoff was selected based on a previous study showing that a threshold of ≥4 biomarkers achieved 100% sensitivity for identifying hypervirulent strains while allowing inclusion of those with a broader hypervirulence-associated biomarker profile (7).

### Statistical analysis

All statistical analyses were conducted using Stata version 19.0. Diagnostic performance, including sensitivity, specificity, positive likelihood ratio, negative likelihood ratio, and accuracy, was calculated with 95% confidence intervals (*CIs*). Paired comparisons of sensitivity and specificity between the two assays were performed using McNemar’s exact test.

## RESULTS

### Species characteristics, hypervirulent status, and *ter* gene carriage

**Fig 2:**
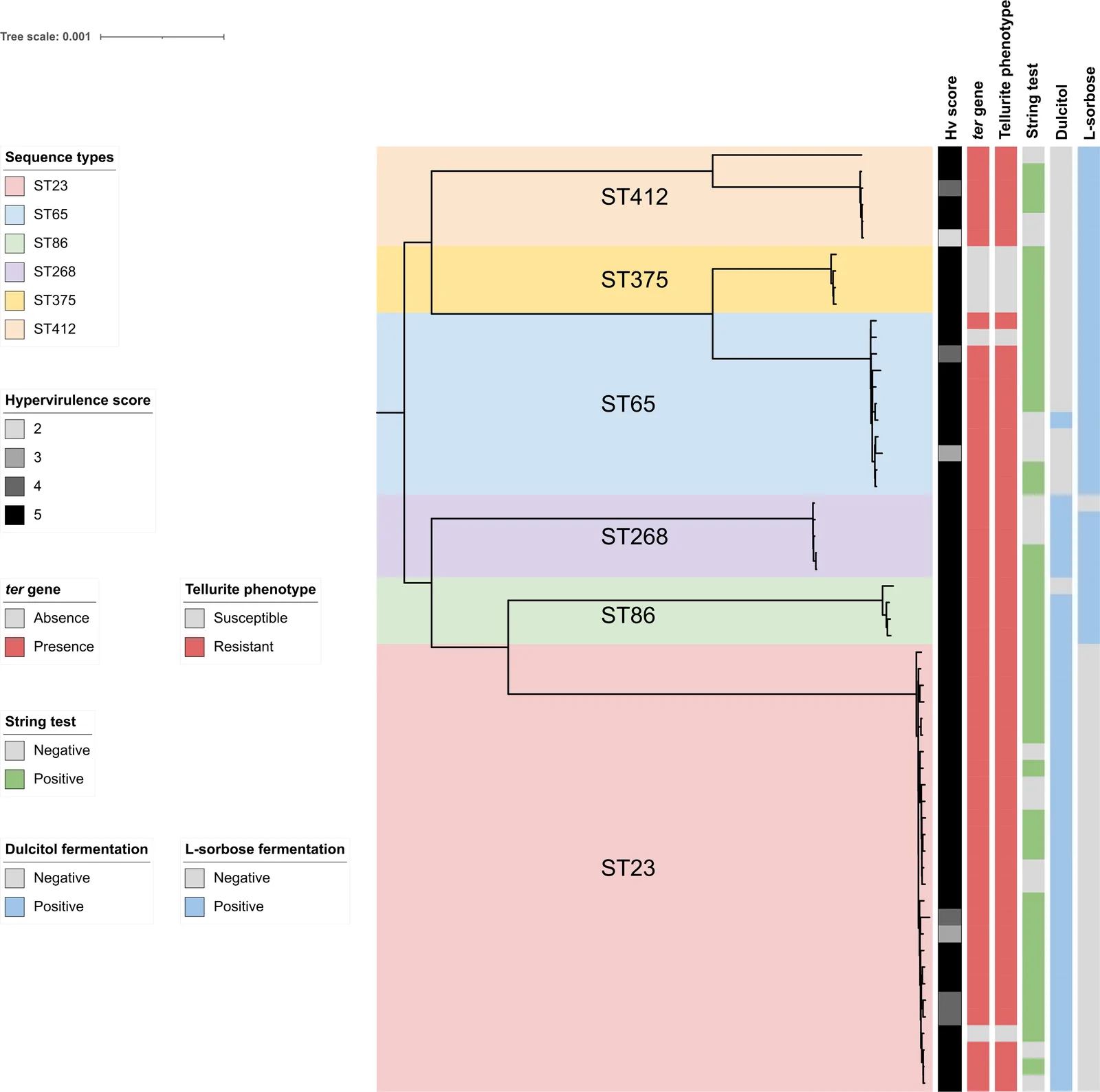
Phylogenetic clustering of major sequence types associated with hypervirulent *Klebsiella pneumoniae* and their genotypic and phenotypic traits. A phylogenetic tree was constructed from core-genome single-nucleotide polymorphism (SNP) alignments of 57 *K. pneumoniae* strains representing major hypervirulent lineages and sequence types (STs). Strains were annotated with ST, hypervirulence score (Hv score), *ter* gene carriage, tellurite phenotype, string test result, dulcitol fermentation, and l-sorbose fermentation. Sequence types are indicated by a colored strip, and Hv score is shown as a grayscale strip. Hv score was defined as the number of virulence-associated genes detected among *iucA*, *iroB*, *peg-344*, *rmpA*, and *rmpA2*. For tellurite phenotype, visible growth on MacConkey agar supplemented with potassium tellurite at 4 μg/mL was interpreted as resistant, whereas no visible growth was interpreted as susceptible. Branch lengths are proportional to the genetic distances inferred from the core-genome SNP alignment. The tree was midpoint-rooted for visualization only.

Among the 193 study strains, the species distribution was *K. pneumoniae* (*n* = 152), *K. variicola* (*n* = 29), and *K. quasipneumoniae* (*n* = 12) (Table 1). Overall, 68 strains (35.2%) met the study definition of hypervirulence, comprising *K. pneumoniae* (*n* = 67) and *K. quasipneumoniae* (*n* = 1) (Table 1). Among hypervirulent *K. pneumoniae* strains, ST23 predominated (*n* = 26), followed by ST65 (*n* = 10), ST268 (*n* = 5), ST412 (*n* = 5), ST86 (*n* = 4), and ST375 (*n* = 4) (Fig. 2).

**TABLE 1:** Hypervirulence status and *ter* gene carriage by species among 193 strains of the *Klebsiella pneumoniae* species complex^*a*^

Characteristic	*K. pneumoniae* (*n* = 152)	*K. variicola* (*n* = 29)	*K. quasipneumoniae* (*n* = 12)	Total (*n* = 193)
Hypervirulent, *n* (%)	67 (44.1)	0 (0.0)	1 (8.3)	68 (35.2)
*ter*-positive, *n* (%)	68 (44.7)	4 (13.8)	2 (16.7)	74 (38.3)

^*a*^ Data are shown as *n* (%). Percentages are based on the number of strains within each species or group. Hypervirulent strains were defined as those carrying ≥4 of the five biomarkers (*iucA*, *iroB*, *peg-344*, *rmpA, *and *rmpA2*).

The presence of *ter* genes was identified in 74 strains (38.3%) (Table 1). Of these, 72 carried all detected *ter* genes from *ter*A to *ter*W. One strain lacked *ter*Y and *ter*W among these genes, and one strain carried only *ter*X. Among all 68 hypervirulent strains, 59 (86.8%) were *ter*-positive (Table 2). Hypervirulent *ter*-negative strains were predominantly ST375 (four out of four strains), with only single strains identified in ST11, ST23, ST65, ST66, and ST380.

**TABLE 2:** *ter* gene carriage within hypervirulent and non-hypervirulent subgroups by species among 193 strains of the *Klebsiella pneumoniae* species complex^*a*^

Characteristic	*K. pneumoniae*	*K. variicola*	*K. quasipneumoniae*	Total
Hypervirulent				
Number of strains, *n*	67	0	1	68
*ter*-positive, *n* (%)	58 (86.6)	NA	1 (100.0)	59 (86.8)
Non-hypervirulent				
Number of strains, *n*	85	29	11	125
*ter*-positive, *n* (%)	10 (11.8)	4 (13.8)	1 (9.1)	15 (12.0)

^*a*^ Data are shown as *n* or *n* (%). Percentages in the *ter*-positive rows are based on the number of strains in the corresponding subgroup. Hypervirulent strains were defined as those carrying ≥4 of the five biomarkers (*iucA*, *iroB*, *peg-344*, *rmpA*, and *rmpA2*). *ter*-positive indicates the presence of one or more *ter* genes. NA, not applicable.

Among the 125 non-hypervirulent strains, 15 were *ter*-positive, including *K. pneumoniae* (*n* = 10), *K. variicola* (*n* = 4), and *K. quasipneumoniae* (*n* = 1) (Table 2). Among the 10 *K. pneumoniae* strains, four carried at least one of the five virulence biomarkers (*iucA*, *iroB*, *peg-344*, *rmpA*, and *rmpA2*) but did not meet the study definition of hypervirulence. Three of these four strains belonged to ST23, ST65, and ST412, which have been associated with hypervirulent lineages.

### Determination of tellurite growth-inhibitory concentrations

Growth-inhibitory concentrations ranged from 16 to 300 μg/mL in *ter*-positive strains (*n* = 13) and from <1 to 4 μg/mL in *ter*-negative strains (*n* = 17) (Table S2). To validate the selected concentration range, an additional 100 strains were randomly sampled and tested at potassium tellurite concentrations of 4 and 8 μg/mL. All *ter*-positive strains (*n* = 16) exhibited growth-inhibitory concentrations >8 µg/mL, whereas all *ter*-negative strains (*n* = 84) exhibited growth-inhibitory concentrations <4 µg/mL. Based on these results, potassium tellurite at 4 μg/mL was used in subsequent experiments.

### Tellurite resistance and adonitol fermentation for hypervirulence screening

MacConkey agar containing potassium tellurite at 4 µg/mL was used to assess the tellurite-resistant phenotype, and adonitol fermentation was evaluated as a supplementary marker for screening. A total of 77 strains (39.9%) showed the tellurite-resistant phenotype. Among these, 72 produced pink colonies and were interpreted as indicating adonitol fermentation, whereas 5 produced white colonies and were interpreted as indicating adonitol non-fermentation (Table 3).

**TABLE 3:** Phenotypic results on tellurite–adonitol MacConkey agar by species and hypervirulence status^*a*^

Characteristic	*K. pneumoniae*	*K. variicola*	*K. quasipneumoniae*	Total
All strains				
Number of strains, *n*	152	29	12	193
Tellurite-resistant phenotype, *n* (%)	70 (46.1)	4 (13.8)	3 (25.0)	77 (39.9)
Adonitol non-fermentation, *n* (%)	1 (1.4)	4 (100.0)	0 (0.0)	5 (6.5)
Hypervirulent				
Number of strains, *n*	67	0	1	68
Tellurite-resistant phenotype, *n* (%)	58 (86.6)	NA	1 (100.0)	59 (86.8)
Adonitol non-fermentation, *n* (%)	0 (0.0)	NA	0 (0.0)	0 (0.0)
Non-hypervirulent				
Number of strains, *n*	85	29	11	125
Tellurite-resistant phenotype, *n* (%)	12 (14.1)	4 (13.8)	2 (18.2)	18 (14.4)
Adonitol non-fermentation, *n* (%)	1 (8.3)	4 (100.0)	0 (0.0)	5 (27.8)

^*a*^ Data are shown as *n* or *n* (%). Percentages in the tellurite-resistant phenotype rows are based on the number of strains in the corresponding group. Percentages in the adonitol non-fermentation rows are based on the number of tellurite-resistant strains in the corresponding group.

The tellurite-resistant phenotype on MacConkey agar containing potassium tellurite at 4 µg/mL closely matched the presence of *ter* genes. Of the 77 tellurite-resistant strains, 73 were *ter*-positive and 4 were *ter*-negative (Table 4). Among the 116 strains without the tellurite-resistant phenotype, only 1 strain was *ter*-positive, and this strain carried only *ter*X.

**TABLE 4:** Relationship between *ter* gene carriage and the tellurite-resistant phenotype among 193 strains of the *Klebsiella pneumoniae* species complex^*a*^

*ter* status	Tellurite-resistant phenotype	Tellurite-susceptible phenotype	Total
*ter*-positive	73	1	74
*ter*-negative	4	115	119
Total	77	116	193

^*a*^ *ter*-positive indicates the presence of one or more *ter* genes. The tellurite-resistant phenotype was defined as visible growth on MacConkey agar supplemented with potassium tellurite at 4 μg/mL.

Among the 72 adonitol-fermenting tellurite-resistant strains, *K. pneumoniae* predominated (*n* = 69), with the remainder being *K. quasipneumoniae* (*n* = 3). The five adonitol-nonfermenting tellurite-resistant strains comprised *K. pneumoniae* (*n* = 1) and *K. variicola* (*n* = 4) (Table 3). Notably, all tellurite-resistant *K. variicola* strains that lacked the five virulence biomarkers (*iucA*, *iroB*, *peg-344*, *rmpA*, and *rmpA2*) were adonitol-nonfermenting, whereas only 1 of 70 tellurite-resistant *K. pneumoniae* strains was adonitol-nonfermenting and all 3 tellurite-resistant *K. quasipneumoniae* strains were adonitol-fermenting.

Among the 68 hypervirulent strains, 59 showed the tellurite-resistant phenotype, whereas 18 of the 125 non-hypervirulent strains also showed this phenotype (Table 3). For diagnostic performance analyses, the five tellurite-resistant non-hypervirulent strains with adonitol non-fermentation were classified as test negative. Using this classification, the tellurite–adonitol MacConkey agar assay yielded a sensitivity of 86.8% (95% CI, 76.4–93.8%) and a specificity of 89.6% (95% CI, 82.9–94.3%). The positive likelihood ratio (LR+) was 8.34 (95% CI, 4.95–14.07), and the negative likelihood ratio (LR−) was 0.15 (95% CI, 0.08–0.27). The overall accuracy was 88.6% (95% CI, 83.3–92.7%) (Table 5).

**TABLE 5:** Diagnostic performance of the tellurite–adonitol MacConkey agar and the string test for identification of hypervirulent strains within the *Klebsiella pneumoniae* species complex^*a*^^,*b*^

Test	Sensitivity, % (95% CI)	Specificity, % (95% CI)	LR+ (95% CI)	LR− (95% CI)	Accuracy, % (95% CI)
Tellurite–adonitol MacConkey agar	86.8 (76.4–93.8)	89.6 (82.9–94.3)	8.34 (4.95–14.07)	0.15 (0.08–0.27)	88.6 (83.3–92.7)
String test	73.5 (61.4–83.5)	72.0 (63.3–79.7)	2.63 (1.92–3.60)	0.37 (0.24–0.55)	72.5 (65.7–78.7)

^*a*^ CI, confidence interval; LR+, positive likelihood ratio; LR−, negative likelihood ratio.

^*b*^ For diagnostic performance analyses, the five tellurite-resistant non-hypervirulent strains with adonitol non-fermentation were classified as test negative.

By comparison, the string test yielded a sensitivity of 73.5% (95% CI, 61.4–83.5%) and a specificity of 72.0% (95% CI, 63.3–79.7%). The positive likelihood ratio (LR+) was 2.63 (95% CI, 1.92–3.60), and the negative likelihood ratio (LR−) was 0.37 (95% CI, 0.24–0.55). The overall accuracy was 72.5% (95% CI, 65.7–78.7%) (Table 5).

In paired comparisons, the tellurite–adonitol assay showed higher sensitivity than the string test among hypervirulent strains, although the difference was not statistically significant (86.8% vs 73.5%, *P* = 0.108). In contrast, specificity was significantly higher for the tellurite–adonitol assay among non-hypervirulent strains (89.6% vs 72.0%, *P* = 0.0005).

### Carbohydrate fermentation patterns among hypervirulent lineages

Based on the preliminary carbohydrate screening described above, dulcitol and l-sorbose were selected for further evaluation on solid medium. Fermentation of dulcitol and l-sorbose was then tested in all 193 strains using MacConkey agar supplemented with each carbohydrate. No carbohydrate fermentation pattern was identified that consistently captured all hypervirulent strains across lineages while excluding non-hypervirulent strains (Table S3). Notably, all 27 ST23 strains showed dulcitol fermentation and l-sorbose non-fermentation (Fig. 2). This phenotype was observed in 42 strains overall (Table S3). By contrast, among other hypervirulent sequence types, ST65, ST375, and ST412 were most often associated with dulcitol non-fermentation and l-sorbose fermentation, whereas ST86 and ST268 were more often associated with fermentation of both carbohydrates (Fig. 2).

## DISCUSSION

While the string test is easy to perform and broadly implemented for detection of hypervirulent *K. pneumoniae* in clinical microbiology laboratories, its suboptimal performance is well documented (10, 11, 26). We show that tellurite-based screening combined with carbohydrate fermentation testing provides a useful phenotypic approach for identifying hypervirulent *K. pneumoniae* and demonstrates higher specificity and numerically higher sensitivity than the string test. Within this approach, adonitol fermentation improved screening performance for hypervirulent *K. pneumoniae*, whereas dulcitol and l-sorbose fermentation patterns supported presumptive lineage identification, including ST23, a representative hypervirulent lineage. These findings indicate that this approach may be useful for screening hypervirulent *K. pneumoniae* and for supporting presumptive identification of selected lineages.

A key strength of this study is that it included the entire *K. pneumoniae* species complex, rather than being limited to *K. pneumoniae* alone. This approach more closely reflects routine clinical practice, in which closely related species may be reported as *K. pneumoniae* and practical phenotypic tools for presumptive recognition of hypervirulence remain limited (1). The clinical value of this method lies in its ability to assess a hypervirulent phenotype within 24 h after detection of a colony suspected to belong to the *K. pneumoniae* species complex. As shown in Fig. 2, string test positivity was observed across multiple major hypervirulent lineages, but string test-negative strains were also present within these lineages, underscoring the limited consistency of this test as a screening marker. Another strength of this study is that the tellurite-based approach achieved significantly higher specificity than the string test while maintaining high sensitivity for hypervirulent strains. Although the present method requires an additional day of incubation compared with the string test, interpretation of the tellurite-resistant phenotype and carbohydrate fermentation patterns is relatively straightforward and may be more reproducible.

In this study, we defined hypervirulent strains as those carrying at least four of the five virulence-associated genes, based on a previous study in which ≥4 biomarkers identified hypervirulent strains with 100% sensitivity (7). This more inclusive cutoff captured strains with a broader hypervirulence-associated biomarker profile. The hypervirulent strains identified by this definition were distributed mainly among sequence types previously associated with hypervirulence, including ST23, ST65, ST86, ST375, ST268, and ST412 (26). Among these, ST23 was the most represented, consistent with prior studies (1, 26). In addition, some *ter*-positive strains that did not meet our definition of hypervirulence belonged to sequence types associated with hypervirulent lineages, suggesting that the *ter* gene cluster may be more closely linked to hypervirulent lineages than reflected by our virulence gene definition. Previous studies have reported associations between *ter*W and hypervirulence-associated lineages, particularly ST23, ST65, and ST86 (12). In our more detailed genome analysis, carriage of all detected *ter* genes from *ter*A to *ter*W was strongly associated with hypervirulent strains. Notably, ST375 lacked detectable *ter* genes, also consistent with previous reports (12).

The tellurite-resistant phenotype observed in this tellurite-based assay correlated well with the presence of *ter* genes, supporting the validity of the selected tellurite concentration of 4 μg/mL established in the pilot analysis. This finding was consistent with previous reports using MacConkey-potassium tellurite medium to detect *ter*W-positive strains (12, 15), and the selected concentration also fell within the previously reported range. Only a small number of strains without detectable *ter* genes exhibited a resistant phenotype at 4 μg/mL, and the strain carrying only *ter*X was tellurite susceptible. Taken together, these findings further support the relevance of the *ter* gene cluster to phenotypic tellurite resistance under the present experimental conditions. However, hypervirulent strains lacking the *ter* gene cluster, exemplified by ST375, were not detected by the tellurite-based assay alone, representing an important limitation of this method.

An important finding of this study was that tellurite-based screening identified *ter*-positive, non-hypervirulent *K. variicola* strains, which may result in false-positive presumptive identification of hypervirulent *K. pneumoniae*. Although previous studies have shown that adonitol non-fermentation alone cannot completely discriminate between *K. variicola* and *K. pneumoniae* (19, 27), incorporating adonitol non-fermentation into the tellurite-based screening assay helped reduce these false-positive results and improved the diagnostic performance of the assay. *K. variicola* can be accurately identified by matrix-assisted laser desorption ionization–time of flight mass spectrometry (MALDI-TOF MS) when updated databases or web-based identification tools are available (28). In laboratories without access to MALDI-TOF MS or such updated identification resources, this phenotype-based approach may be particularly useful.

No single carbohydrate fermentation pattern reliably distinguished hypervirulent strains across lineages from non-hypervirulent strains. Nonetheless, dulcitol fermentation and l-sorbose non-fermentation were observed in all ST23 strains in this study. Although this phenotype was not specific to ST23, its combination with the tellurite-resistant phenotype enriched for hypervirulent strains, most of which belonged to ST23, and may therefore support presumptive identification of this lineage. This observation is consistent with a previous study examining the metabolic capabilities of genetically diverse *K. pneumoniae* strains, which showed that metabolic profiles correlated closely with MLST-defined clones (29). Thus, dulcitol and l-sorbose fermentation patterns may also serve as complementary phenotypic markers for selected hypervirulent lineages.

This study has several limitations. First, all strains were collected in Japan, and the generalizability of these findings to hypervirulent strains circulating in other geographic regions remains uncertain. Nevertheless, this study included a relatively large number of hypervirulent strains across a range of lineages, which would likely have been more difficult to assemble in a consecutive clinical cohort. Second, although our definition of hypervirulent strains was based on virulence-associated genes supported by previous studies, it may not fully capture the biological and phenotypic heterogeneity of hypervirulence. Third, fermentation testing did not provide definitive classification in all cases, limiting its utility as a standalone approach.

In conclusion, this tellurite-based approach offers a simple and practical culture-based method for screening hypervirulent *K. pneumoniae*. Incorporation of adonitol fermentation may further enhance diagnostic performance, while dulcitol and l-sorbose fermentation patterns may assist in presumptive identification of selected hypervirulent lineages in clinical microbiology laboratories.

## Data Availability

The genome sequencing data are available under the umbrella BioProject accession number PRJDB42677. This includes data newly deposited in this study as well as data previously deposited under other BioProjects. BioSample accession numbers for all strains are listed in Table S3.

## SUPPLEMENTAL MATERIAL

Supplemental tables spectrum.01838-26-s0001.pdf Tables S1 and S2.

Table S3 spectrum.01838-26-s0002.xlsx Genotypic and phenotypic characteristics of all 193 strains of *Klebsiella pneumoniae* species complex included in the study.

ASM does not own the copyrights to Supplemental Material that may be linked to, or accessed through, an article. The authors have granted ASM a non-exclusive, world-wide license to publish the Supplemental Material files. Please contact the corresponding author directly for reuse.
