# Supplementary material for: Phenotypic screening of hypervirulent Klebsiella pneumoniae using tellurite resistance and carbohydrate fermentation testing
Source: Microbiol Spectr. 2026 Aug 17;14(10):e01838-26. doi: 10.1128/spectrum.01838-26 (PMC13689481; doi:10.1128/spectrum.01838-26)
Supplement: Supplementary file 1 [file spectrum.01838-26-s0001.pdf]

**Supplementary Table 1. Reference genomes.**

| Species | Taxonomy ID | NCBI RefSeq assembly | BioProject | BioSample ID | Strain |
|---|---|---|---|---|---|
| *Klebsiella pneumoniae* | 1125630 | GCF_000240185.1 | PRJNA78789 | SAMN02602959 | HS11286 |
| *Klebsiella quasipneumoniae* | 1463165 | GCF_020099175.1 | PRJNA231221 | SAMN21218859 | FDAARGOS_1503 |
| *Klebsiella variicola* | 2590157 | GCF_020525545.1 | PRJNA768294 | SAMN22024815 | F2R9T |

**Supplementary Table 2. Distribution of potassium tellurite growth-inhibitory concentrations according to *ter* gene carriage in the pilot set.**

| Growth-inhibitory concentration, µg/mL | *ter*-positive (n = 13) | *ter*-negative (n = 17) |
|---|---|---|
| <1 | 0 | 15 |
| 2 | 0 | 1 |
| 4 | 0 | 1 |
| 8 | 0 | 0 |
| 16 | 1 | 0 |
| 50 | 2 | 0 |
| 100 | 2 | 0 |
| 300 | 8 | 0 |

The tellurite growth-inhibitory concentration was defined as the lowest concentration that completely inhibited visible growth on MacConkey agar supplemented with potassium tellurite.
